# Supplementary material for: Accurate prediction of protein function using statistics-informed graph networks
Source: Nat Commun. 2024 Aug 4;15:6601. doi: 10.1038/s41467-024-50955-0 (PMC11297950; doi:10.1038/s41467-024-50955-0)
Supplement: Supplementary file 1 — Supplementary Information [file 41467_2024_50955_MOESM1_ESM.pdf]

Supplementary materials
Accurate prediction of protein function using statistics-informed graph networks

1 PhiGnet architecture

Input: one-hot encoding sequence: (batch, L, 26), EVCs, RCs, ESM-1b_feature.

GCN_inputs:

sequence_embedding = Dense (dim = 1024, use_bias = False) (one-hot encoding sequence)

ESM-1b_embedding = Dense (dim = 1024, use_bias = True) (ESM-1b_feature)

GCN_inputs = RELU (sequence_embedding, ESM-1b_embedding)

GCN Architecture:

GCN-1: three GCN layers with 512 dim and ELU activation, GCN_inputs as the input of GCN-1 and EVCs as the adjacency matrix of each layer.

GCN-2: three GCN layers with 512 dim and ELU activation, GCN_inputs as the input of GCN-2 and RCs as the adjacency matrix of each layer.

Output:

output = Concatenate(GCN-1, GCN-2)

output = Sum Pooling()

output = Dense(dim = 1024, activation=RELU)

output = Dropout()

output = Dense()

output = Softmax()

2 Scoring metrics

The precision that measures the predictive accuracy of each method is defined as follows:

$$\text{p(t)} = \frac{1}{m(t)} \sum_{i=1}^{m(t)} \frac{\sum_f I(f \in P_i(t) \wedge f \in T_i)}{\sum_f I(f \in P_i(t))}, \tag{S1}$$

where t is a threshold ($t \in [0,1]$) with an increment of 0.01, f is a label of a single function annotation (EC number or GO term), $m(t)$ is the number of proteins with prediction scores not less than the threshold t for predicting at least one function class, $P_i(t)$ is the set of predicted annotations of the ith protein at threshold t, T_i denotes all the function classes of the ith protein, and $I(\cdot)$ is the indicator function, returns 1 when the condition is true, otherwise returns 0.

The recall is used to measure relevant information that is successfully retrieved and defined as follows:

$$\text{r(t)} = \frac{1}{N_T} \sum_{i=1}^{N_T} \frac{\sum_f I(f \in P_i(t) \wedge f \in T_i)}{\sum_f I(f \in T_i)}, \tag{S2}$$

where N_T is the total number of the test proteins. We compute the F-score using the values of precision $p(t)$ and recall $r(t)$ under different thresholds $t \in [0,1]$ and achieve the maximum of the F-score, F_{max} score. AUPR is computed under the macro-averaging of Precision-Recall curves.

We employ Matthew's correlation coefficient (MCC) that evaluates the correlation between prediction and the ground truth to compare the performances of different methods, and it comprehensively considers the four basic evaluation indexes in the confusion matrix. Its definition is presented as follows:

$$\text{MCC} = \frac{TP \times TN - FP \times FN}{\sqrt{(TP+FP)(TP+FN)(TN+FP)(TN+FN)}} \tag{S3}$$

TP is the number of true positives in the situation of prediction under threshold $t \in (0,1)$, similarly, FP is the number of false positives, TN is the number of true negatives, and FN is the number of false negatives, we computed the average MCC across the test set for all compared methods to compare.

3 Functional annotation at the residue level

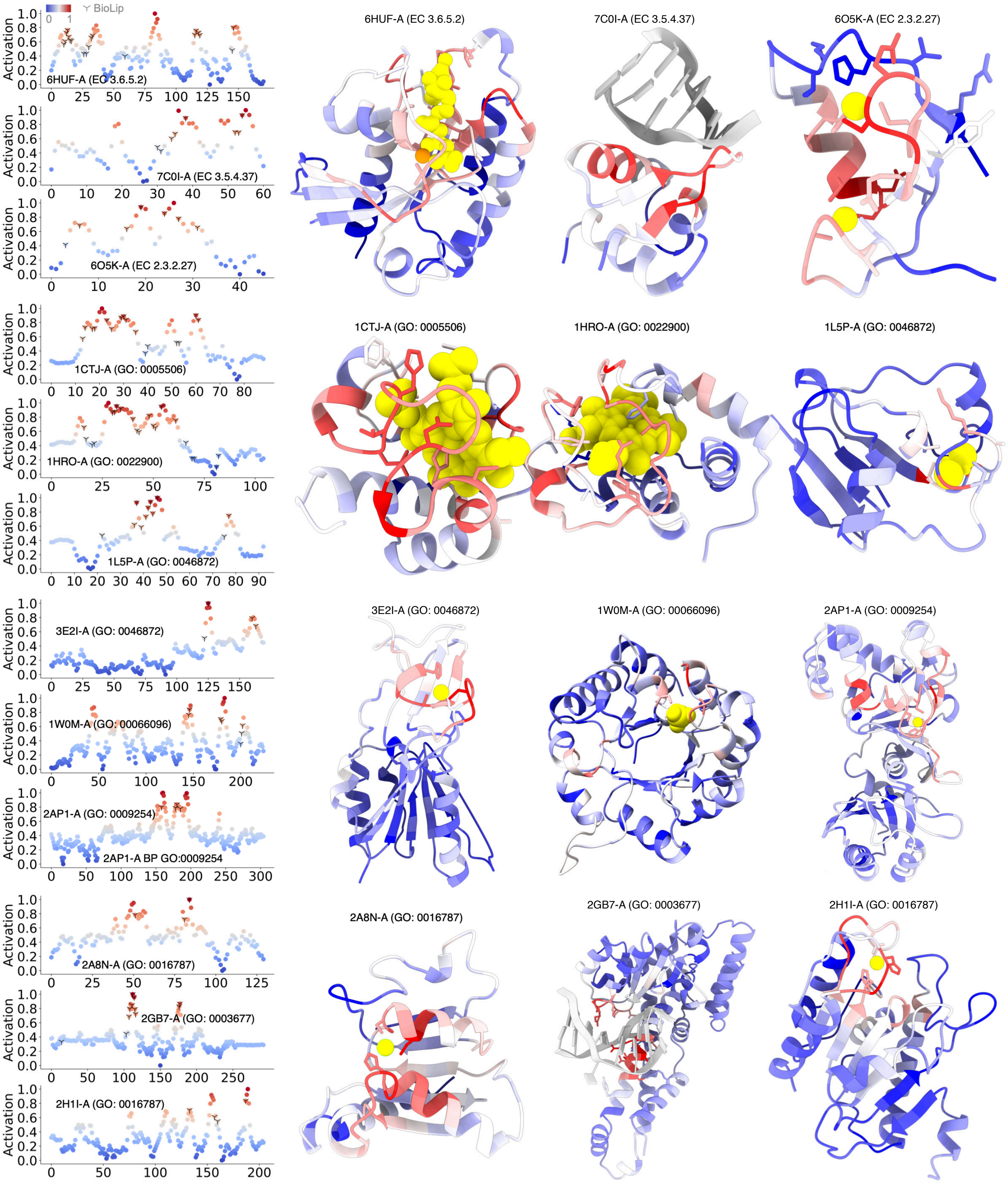

Figure S1. PhiGnet annotates protein function at the residue level. Source data are provided as a Source Data file.

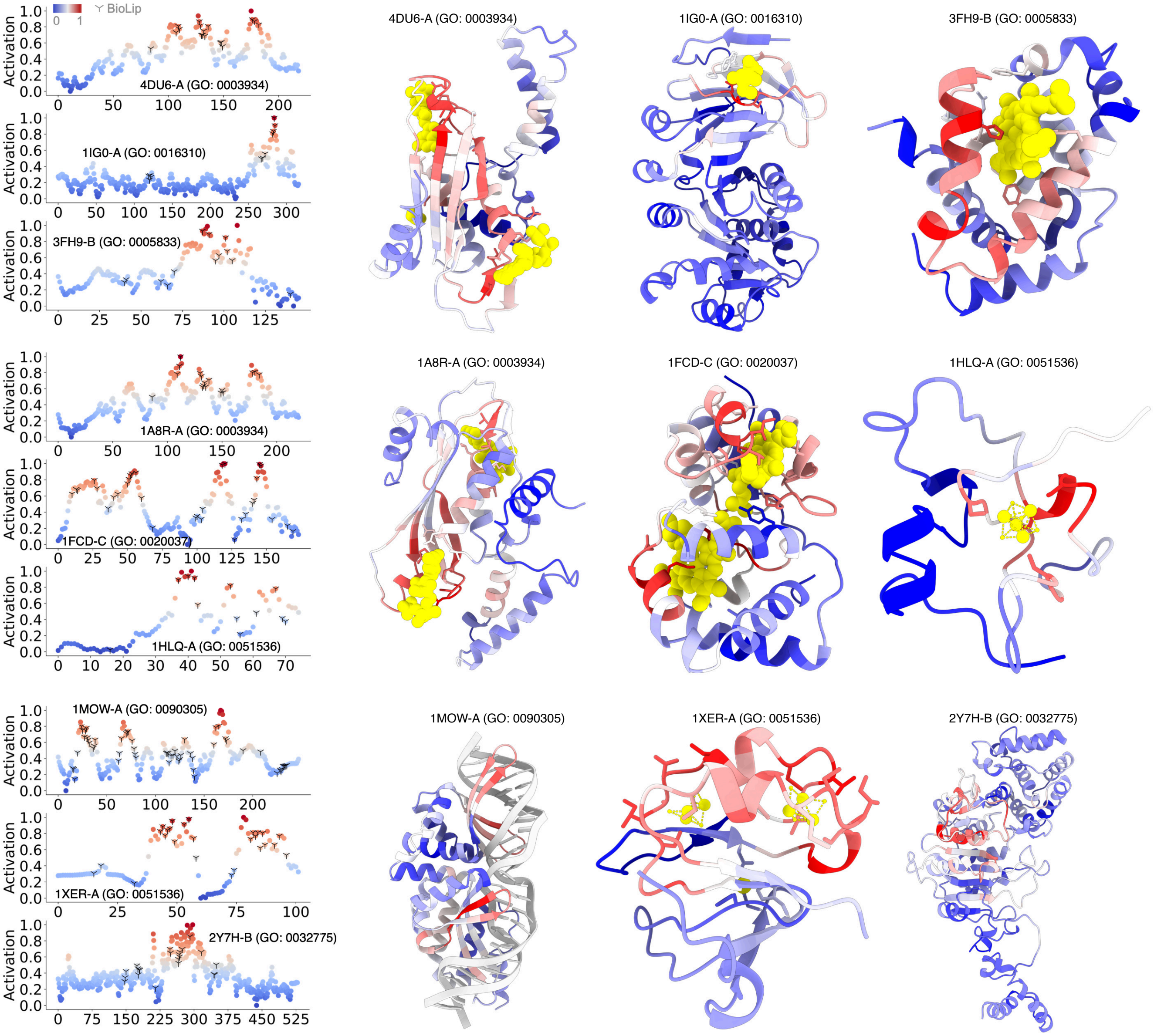

Figure S1. PhiGnet annotates protein function at the residue level (continuous). Source data are provided as a Source Data file.

We screened about 5,181 proteins in the test sets of GO terms and EC numbers using three rules: (1) a protein has a crystal structure; (2) the protein exhibits either ligand, ion, or DNA binding; and (3) the binding sites are also recorded in the BioLip database. Of these proteins, we focused on nine examples to demonstrate the mapped activation scores on their tertiary structures.

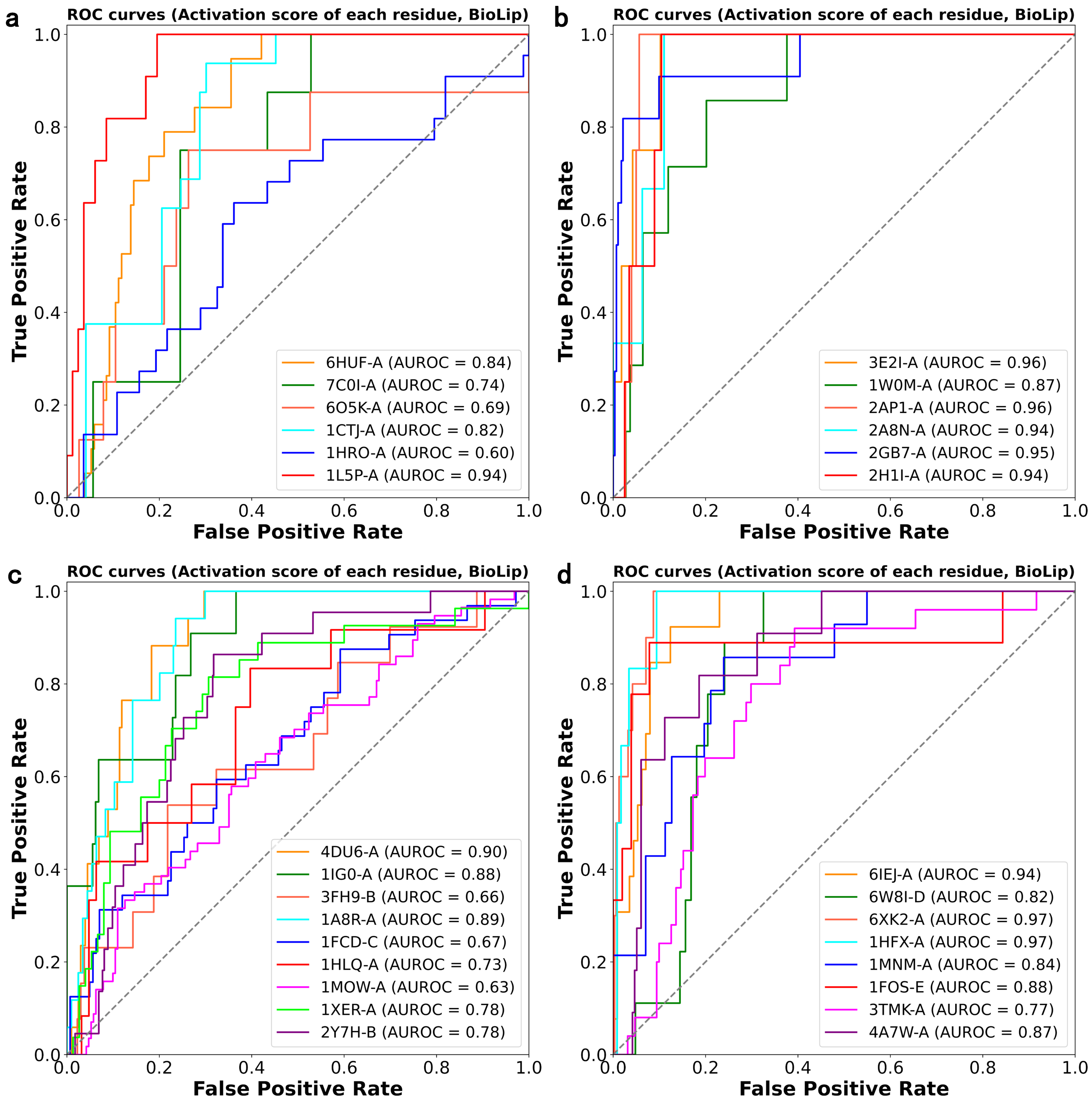

Figure S2. The overlap between activation scores of residues and functional sites obtained from BioLip for the test proteins. The activation scores of residues are computed using the grad-CAM method[1], compared to that of the semi-manually cured database BioLip. Source data are provided as a Source Data file.

4 Performance analysis of PhiGnet

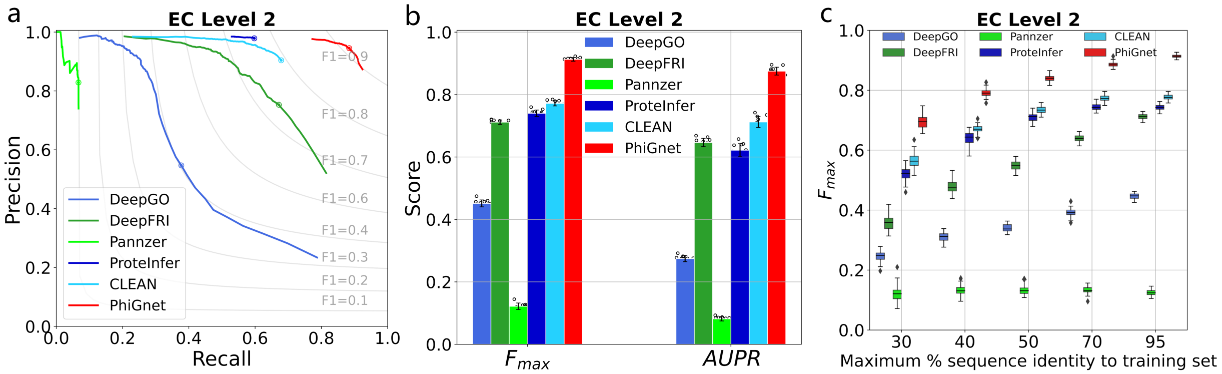

Figure S3. The performance comparison between DeepGO, DeepFRI, Pannzer, ProteInfer (Ensembled CNNs), CLEAN, and PhiGnet for predicting EC numbers at Level 2. The distribution of F_{max} scores and AUPR scores across 10 bootstrap iterations. The error bars denote the standard deviation of the mean. The boxplots present the median as the center line of 50 bootstrap iterations, with upper and lower edges indicating the interquartile range; whiskers represent 0.5 times the interquartile range. Source data are provided as a Source Data file.

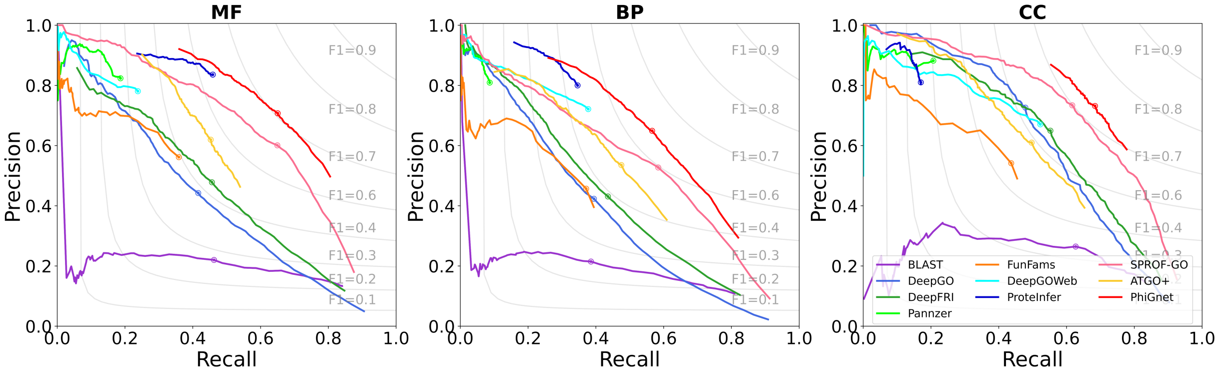

Figure S4. Comparison over test set of ≤30% sequence identity to the training set for predictions of GO terms. Source data are provided as a Source Data file.

Table S1. Comparison among the state-of-the-art methods over GO terms.

Method	F_{max}			AUPR		
	MF	BP	CC	MF	BP	CC
BLAST	0.367	0.349	0.432	0.150	0.072	0.155
FunFams	0.553	0.488	0.535	0.491	0.324	0.308
DeepGO	0.480	0.459	0.616	0.101	0.071	0.154
DeepFRI	0.570	0.526	0.690	0.451	0.302	0.361
PANNZER†	0.301	0.150	0.332	0.470	0.209	0.309
DeepGOWeb†	0.396	0.495	0.621	0.165	0.101	0.157
ProteInfer	0.712	0.602	0.371	0.552	0.358	0.301
SPROF-GO†	0.661	0.585	0.716	0.288	0.291	0.417
ATGO+	0.59	0.539	0.569	0.59	0.191	0.367
PhiGnet	0.811	0.751	0.824	0.804	0.647	0.636

†Results were collected from PANNZER, DeepGOWeb, and SPROF-GO web-servers over the test proteins.

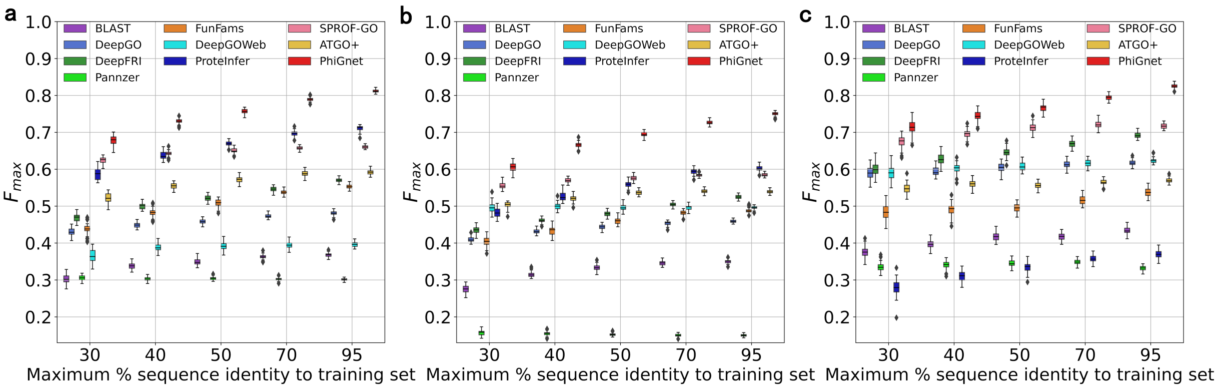

Figure S5. The robustness of PhiGnet in predicting GO terms. At the different levels of sequence identity to the training set, the robustness of different methods are compared over three branches of GO terms, including (a) MF, (b) BP, and (c) CC. The boxplots present the median as the center line of 50 bootstrap iterations, with upper and lower edges indicating the interquartile range; whiskers represent 0.5 times the interquartile range. Source data are provided as a Source Data file.

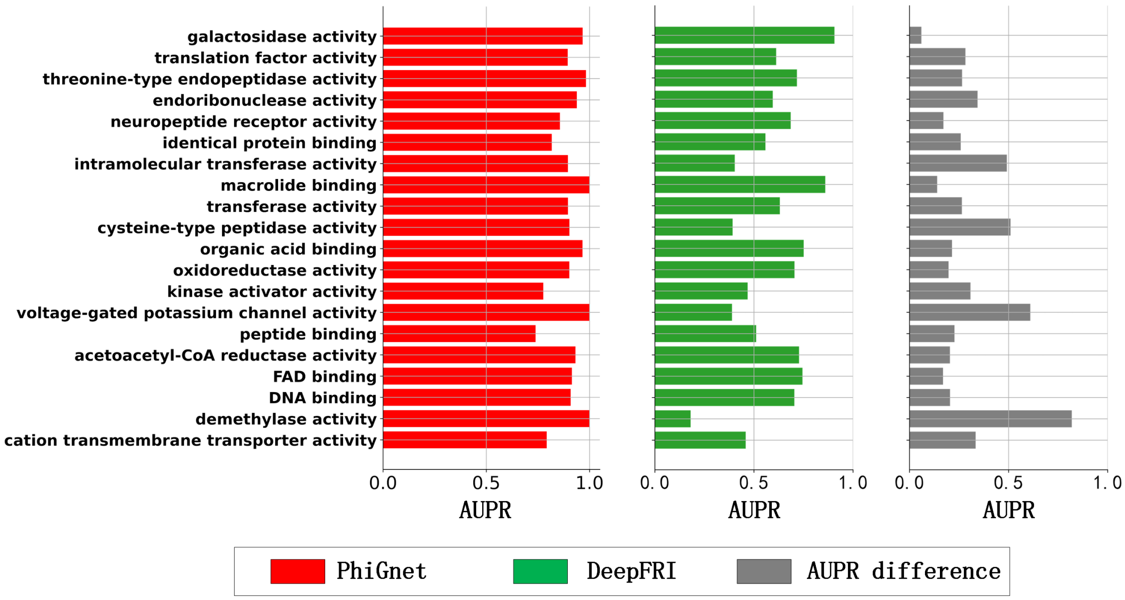

Figure S6. AUPR difference between PhiGnet and DeepFRI over single GO term. Source data are provided as a Source Data file.

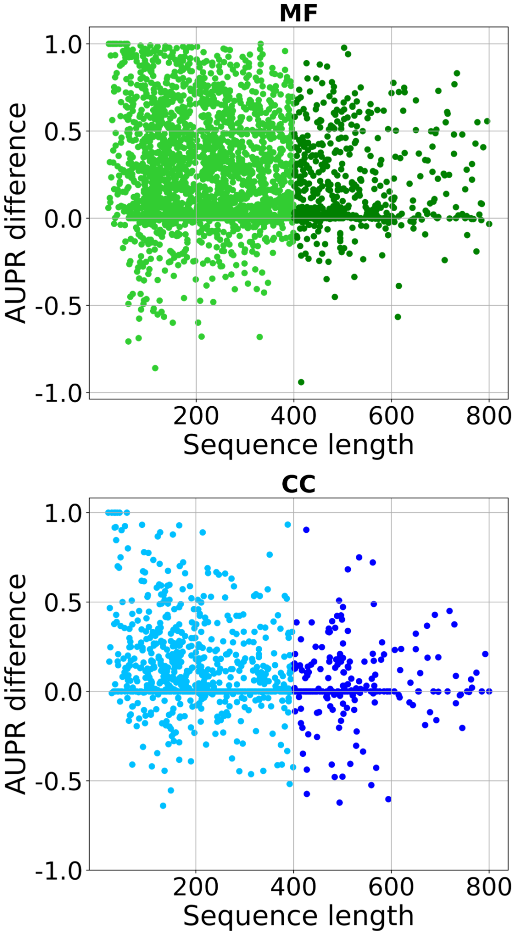

Figure S7. **The differences in AUPR between PhiGnet and DeepFRI across proteins with different numbers of residues.** Source data are provided as a Source Data file.

5 Hyper-parameter tuning

In this study, we employed a threshold to mitigate potential noise arising from coevolution or weak couplings between pairwise residues. To obtain the threshold, we varied its values that were applied to the normalized EVCs/RCs and compared the model's performances with different values of the threshold (see Figure S8). Among our preliminary demonstrations, the PhiGnet model exhibited the best performance with a threshold of 0.2. In all the training and tests, we chose the threshold of 0.2 for both EVCs and RCs.

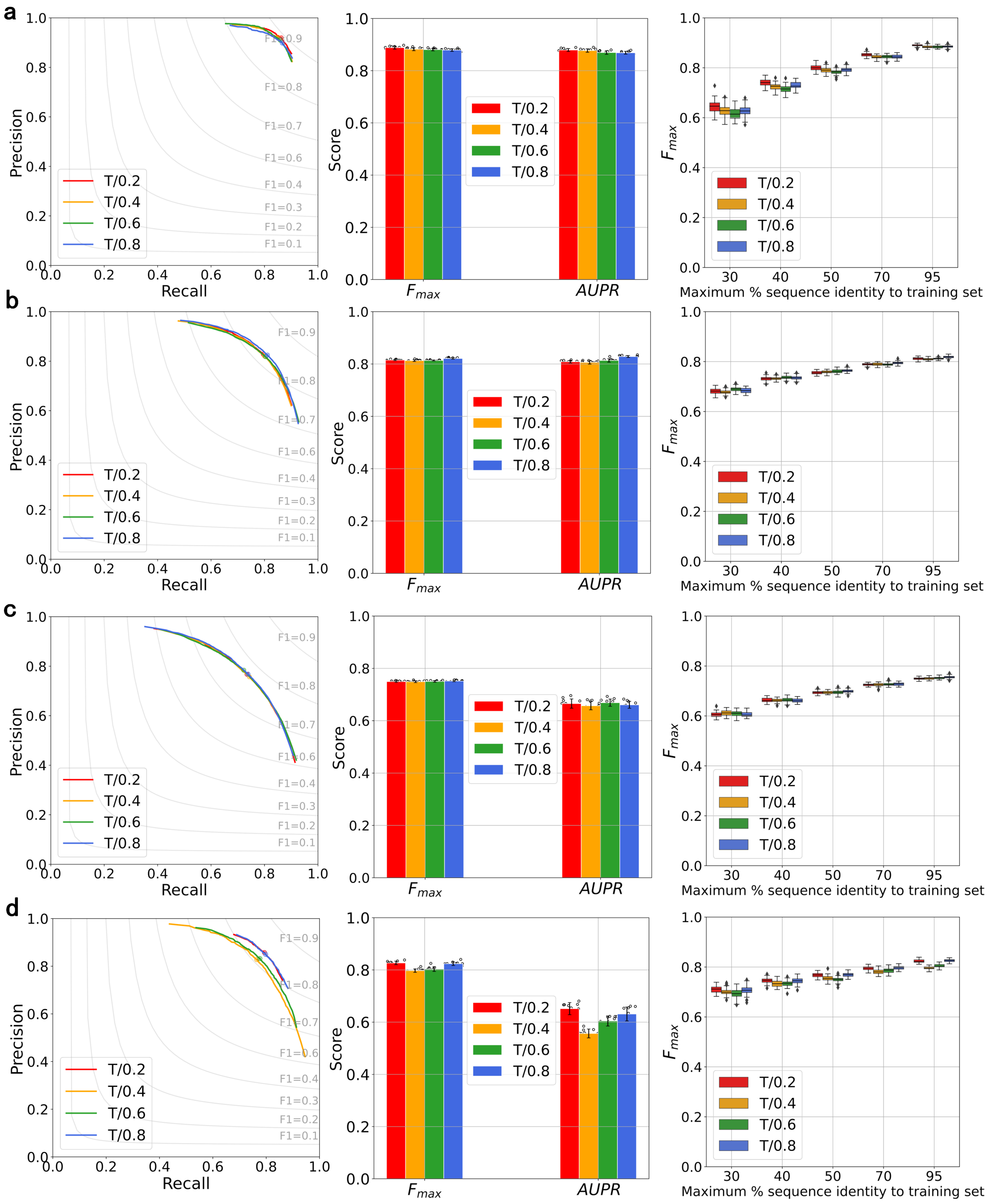

Figure S8. The effect of normalized EVCs and RCs on PhiGnet performance of different thresholds. (a), (b), (c), and (d) present the influence of different thresholds (T$\in$ $(0, 1)$) of EVCs and RCs on the PhiGnet's performance over EC, MF, BP, and CC, respectively. The distribution of F_{max} scores and AUPR scores across 10 bootstrap iterations. The error bars denote the standard deviation of the mean. The boxplots present the median as the center line of 50 bootstrap iterations, with upper and lower edges indicating the interquartile range; whiskers represent 0.5 times the interquartile range. Source data are provided as a Source Data file.

6 Evolutionary information advances PhiGnet

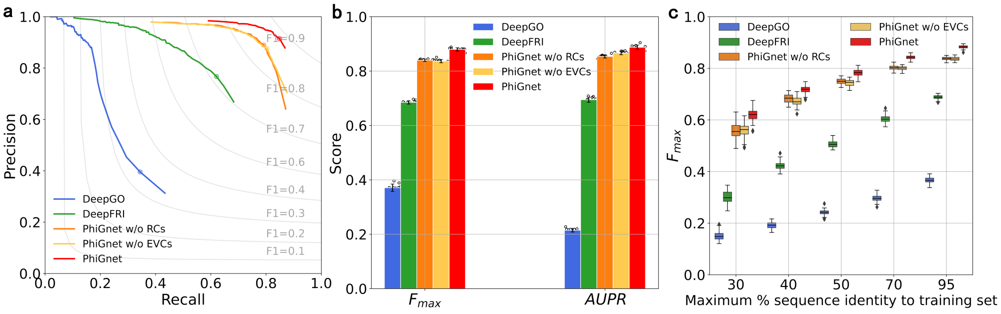

Figure S9. Comparison between different methods in prediction of EC numbers. (a) Comparison of precision-recall among DeepGO, DeepFRI, and PhiGnet with/without evolutionary information. (b) Comparison of different methods over protein-centric F_{max} and terms-centric AUPR. (c) Robustness of different methods over predictions of EC numbers. The distribution of F_{max} scores and AUPR scores across 10 bootstrap iterations. The error bars denote the standard deviation of the mean. The boxplots present the median as the center line of 50 bootstrap iterations, with upper and lower edges indicating the interquartile range; whiskers represent 0.5 times the interquartile range. Source data are provided as a Source Data file.

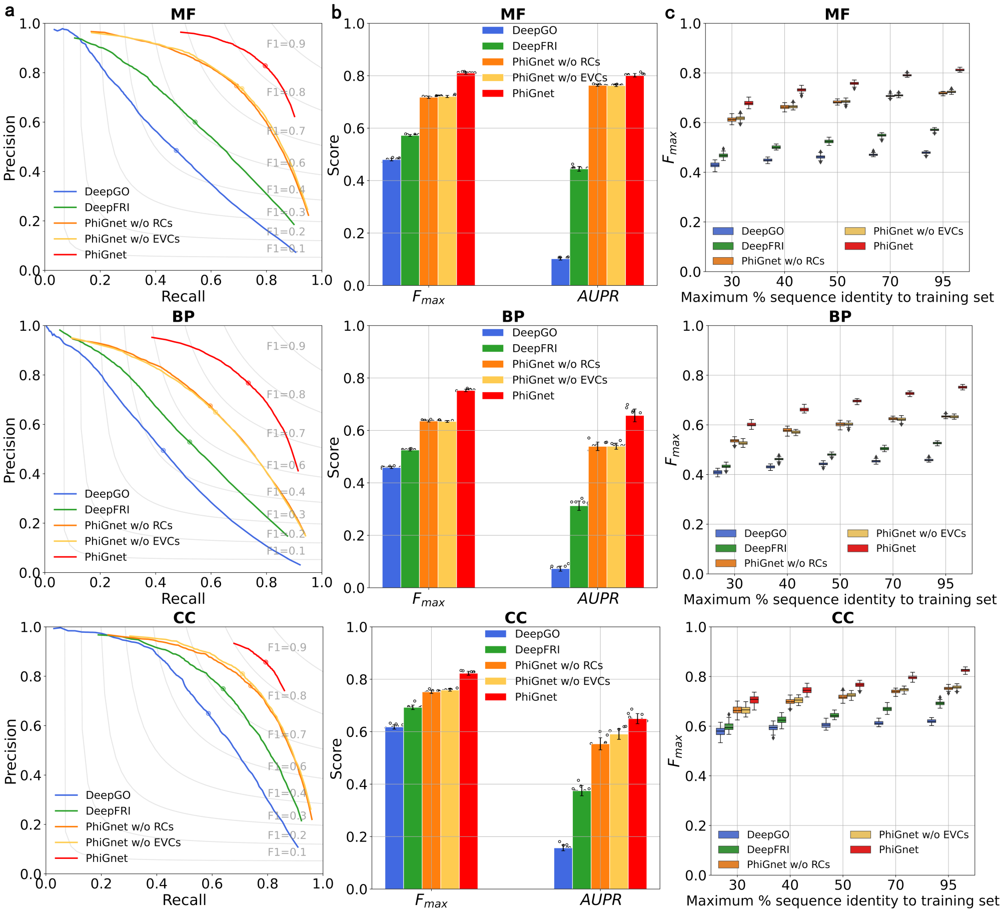

Figure S10. Comparison between different methods in predictions of GO terms. The distribution of F_{max} scores and AUPR scores across 10 bootstrap iterations. The error bars denote the standard deviation of the mean. The boxplots present the median as the center line of 50 bootstrap iterations, with upper and lower edges indicating the interquartile range; whiskers represent 0.5 times the interquartile range. Source data are provided as a Source Data file.

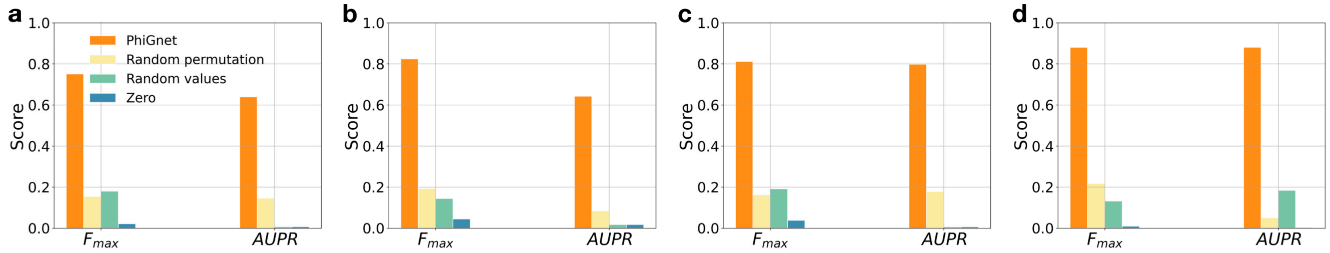

Figure S11. Performance of PhiGnet with different adjacency matrices over the function categories of (a) BP, (b) CC, (c) MF, and (d) EC. Source data are provided as a Source Data file.

7 Comparison on CAFA3

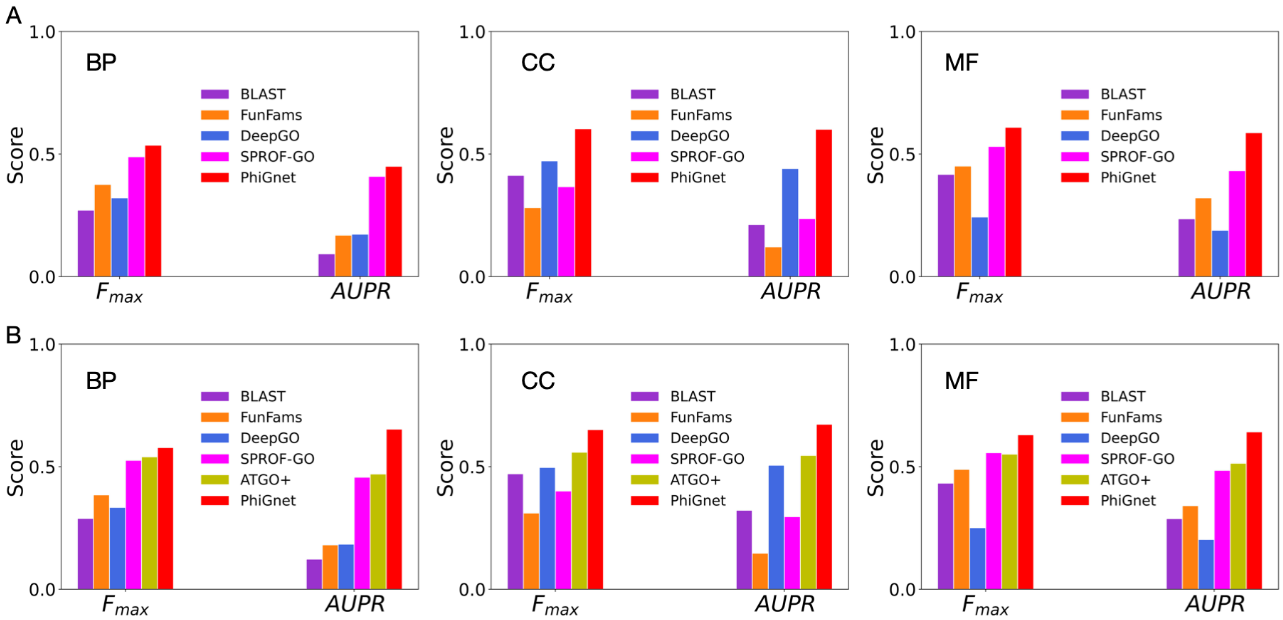

Figure S12. Comparison among the state-of-the-art methods over the CAFA3 dataset (A) with sequence identity less than 60% and (B) without redundancy removal. Source data are provided as a Source Data file.

8 Temporal holdout evaluation

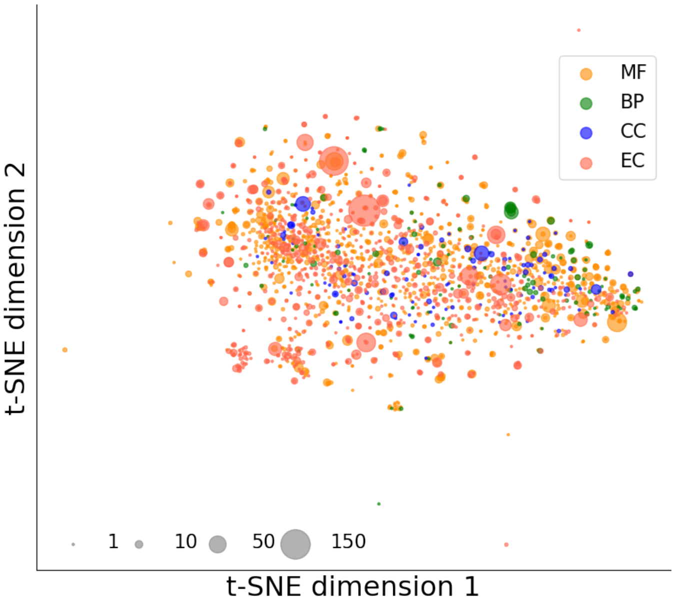

Figure S13. **Clustering proteins in the temporal holdout validation set.** We build a temporal holdout test set were annotated between 1/2022 and 12/2022 (MF: 2,841, BP: 379, CC: 365, EC: 2,795) from the RCSB PDB[3] database. We generated using the t-distributed stochastic neighbor embedding (t-SNE) method. We first computed the Hamming distance between protein sequences as a measure of similarity for clustering purposes. The t-SNE tool was employed to visualize the similarity of the proteins. The positions of the clusters were determined from the two components of the t-SNE outputs. Each point represents a cluster, with the size of each bubble indicating the cluster's size. Source data are provided as a Source Data file.

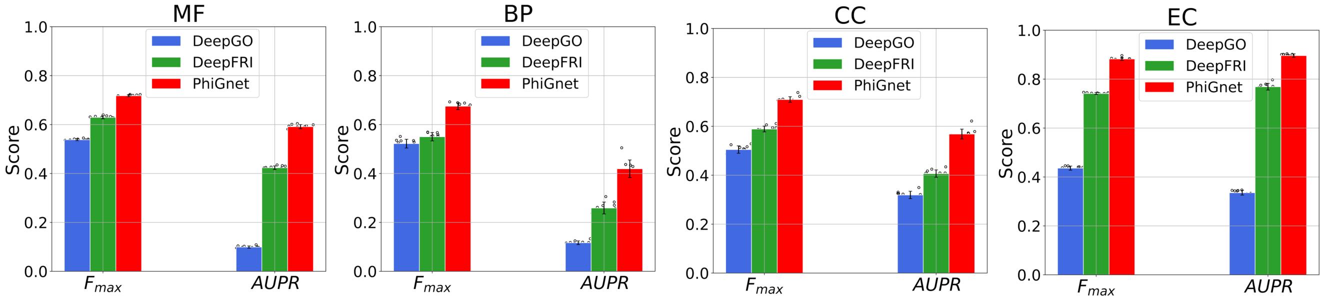

Figure S14. **Comparison among DeepGO, DeepFRI and PhiGnet on a temporal holdout validation.** PhiGnet achieved F_{max} of 0.72, 0.68, 0.71, 0.88 and AUPR of 0.59, 0.42, 0.57, 0.90 for MF, BP, CC, and EC, respectively. DeepFRI had F_{max} of 0.63, 0.55, 0.59, 0.74 and AUPR of 0.42, 0.24, 0.38, 0.77 for the same branches, while DeepGO obtained F_{max} of 0.54, 0.52, 0.50, 0.43 and AUPR of 0.10, 0.11, 0.31, 0.34, respectively. The distribution of F_{max} scores and AUPR scores across 10 bootstrap iterations. The error bars denote the standard deviation of the mean. Source data are provided as a Source Data file.

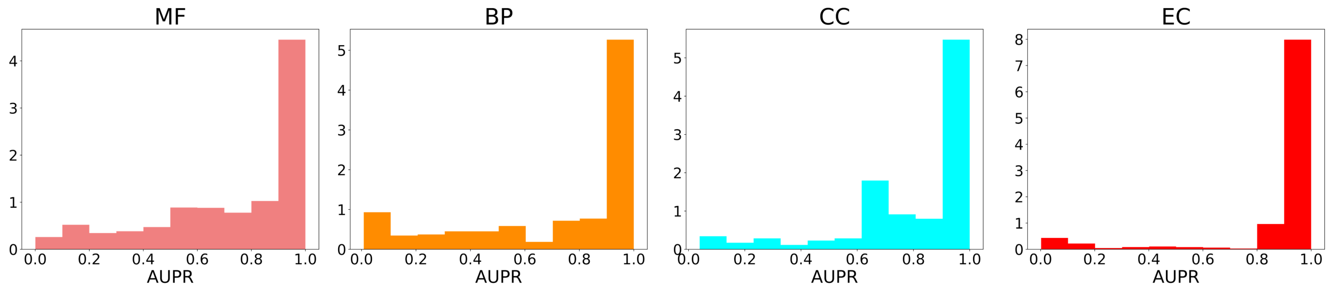

Figure S15. Distributions of AUPR values over the temporal holdout validation set. Source data are provided as a Source Data file.

Table S2. Comparing performances among the state-of-the-art methods for predicting GO terms and EC numbers over proteins released between 30/11/2022 and 7/12/2022 in the RCSB PDB database.

Protein	Annotation	DeepFRI	DeepGOWeb	Pannzer	PhiGnet
7Q9V-A	GO:0004105	0.00	0.29	0.85	0.95
7S5S-A	GO:0008800	0.61	0.50	0.78	1.00
7S5S-A	EC: 3.5.2.6	1.00	–	0.78	1.00
7Y75-A	GO:0005886	0.59	0.68	0.00	0.98
8E0A-A	GO:0032580	0.00	0.00	0.00	0.96
8E0A-A	GO:0006886	0.78	0.00	0.68	0.97
8ESQ-A	GO:0005730	0.00	0.53	0.64	0.92
8F6C-A	GO:0070469	0.73	0.22	0.64	1.00
8GRD-A	GO:0005739	0.43	0.42	0.00	0.92
8H03-A	GO:0005737	0.89	0.75	0.00	1.00
7EWH-A	EC: 1.1.1.37	0.00	–	0.00	0.89
7LCR-A	EC: 3.4.22.69	0.88	–	0.00	0.97
7PCU-A	GO:0003723	0.11	0.50	0.00	1.00
7Q72-A	EC: 2.7.7.19	0.00	–	0.81	0.71
7Q72-A	GO:0005634	0.62	0.48	0.59	1.00
7QXO-A	GO:0004308	0.66	0.50	0.77	0.98
7SUB-A	GO:0004316	0.46	0.00	0.00	0.89
7T0Y-A	GO:0016791	0.50	0.42	0.00	1.00
7T0Y-A	GO:0005977	0.27	0.00	0.75	1.00
7U2K-A	GO:0044877	0.11	0.11	0.56	0.82
7VN3-A	GO:0042597	0.92	0.25	0.67	1.00
7W2A-A	GO:0006508	0.80	0.29	0.61	1.00
7XQN-A	GO:0030060	0.51	0.28	0.80	0.99
7YI7-A	GO:0060055	0.00	0.00	0.00	0.74
7ZYK-A	EC:2.7.11.1	0.66	–	0.69	1.00
7ZYK-A	GO:0005956	0.25	0.18	0.44	0.94
8AGB-A	EC: 2.4.99.-	1.00	–	0.00	1.00
8AGB-A	GO:0004579	0.72	0.17	0.00	1.00
8B6L-A	GO:0016020	1.00	0.66	0.00	1.00

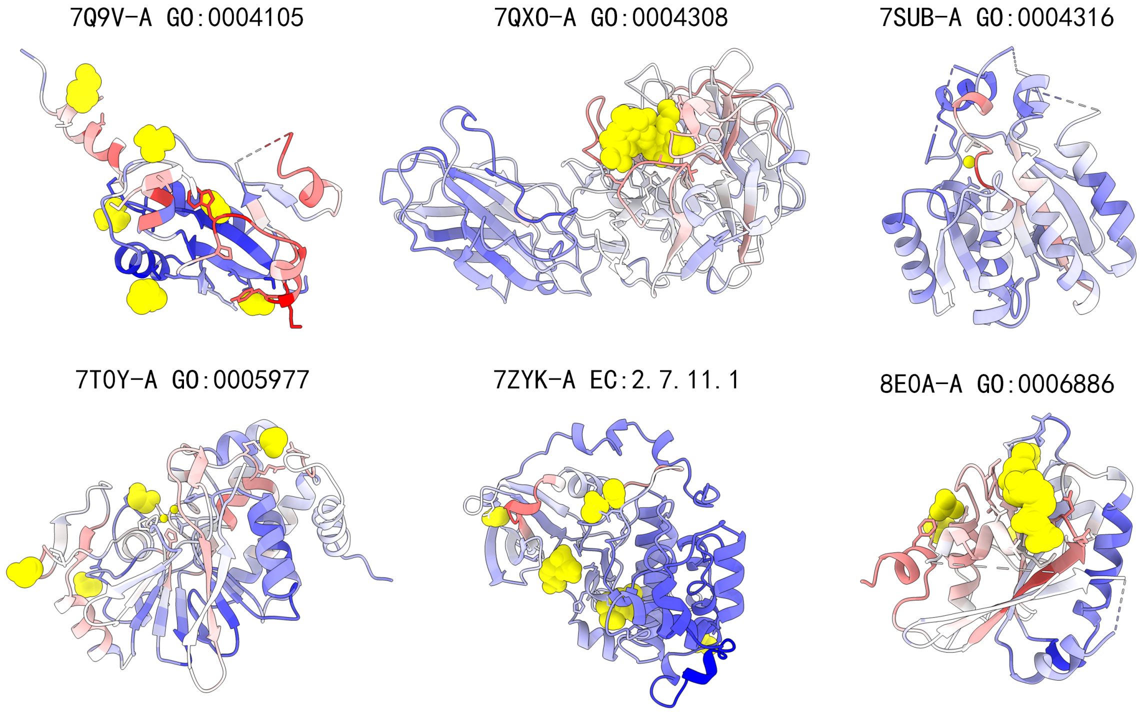

Figure S16. **Mapping activation scores on the tertiary structures of example proteins released between 30/11/2022 and 7/12/2022 in the RCSB PDB database.** Source data are provided as a Source Data file.

9 Similarity between pairwise sequences

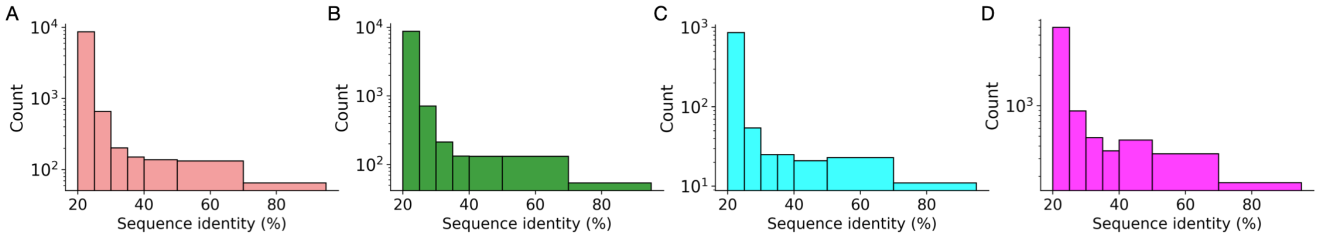

Figure S17. **Pairwise sequence identity in the test sets of (a) MF, (b) BP, (c) CC, (d) EC**. Source data are provided as a Source Data file.

10 The quality of MSAs

We computed the quality of the multiple sequence alignments (MSAs) for the proteins used in both the training and test sets. The distributions of MSAs' quality are illustrated in Figure S18. An MSA with a quality score greater than 1 indicates sufficient diversity of sequences, while a score below 1 implies inadequacy for extracting evolutionary data. In this study, the developed model is constructed based on knowledge extracted from these MSAs. The training of the model achieved a good performance by balancing the information derived from MSAs with quality scores less than 1 (it may be a constant bias), as the distributions of MSAs' quality are similar in both the training and test sets.

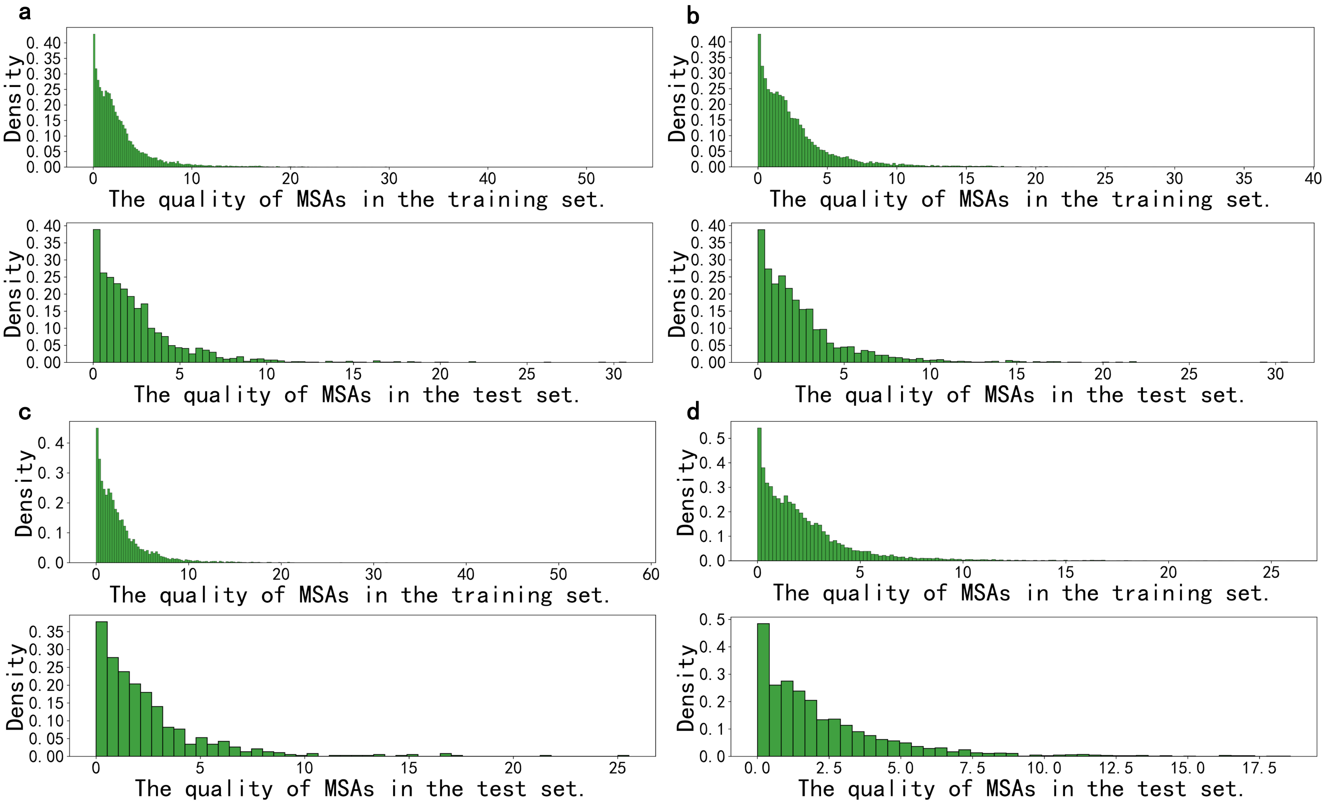

Figure S18. The distributions for the quality of MSAs in the datasets of (a) MF, (b) BP, (c) CC, (d) EC. Source data are provided as a Source Data file.

11 The correlation between the two channels

PhiGnet uses two channels of GCNs, each channel has three GCN layers, using EVCs and RCs as adjacency matrices for the two channels, to model the relationship between a protein sequence and its function.

Here, we ask what differences between the two channels have been learned by the PhiGnet model. We firstly implemented the principal component analysis (PCA)[2] method to extract the top component from the outputs of each GCN layer in either channels, and Pearson correlation coefficient (PCC) of each pair of compared outputs was computed to address the differences between the two channels. Over ten benchmark proteins of more than 400 residues, PCCs were computed (Fig. S19) for comparison. Overall, the PCCs computed over each protein are decreased when the layers get deeper, that is, the deeper the model is, the smaller the PCCs are, indicating each channel puts its attention to learn what it 'cares' more and can extract knowledge at different scales that are results of the differences between EVCs and RCs.

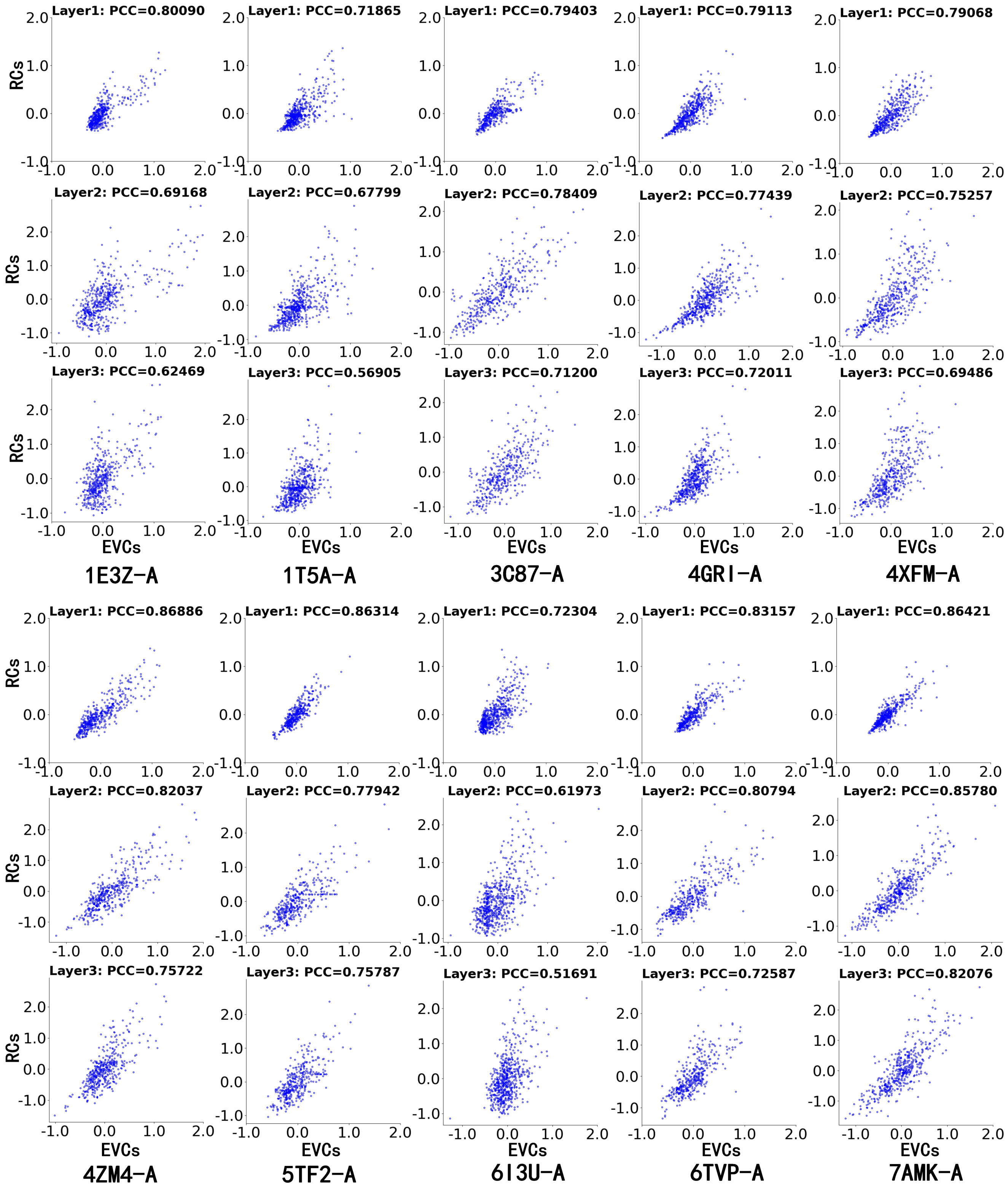

Figure S19. **The degree of correlation between channels of either EVCs or RCs in each GCN layer.** Source data are provided as a Source Data file.

References

1. Selvaraju, R. R. et al. Grad-CAM: Visual explanations from deep networks via gradient-based localization. In Proceedings of the IEEE international conference on computer vision, 618–626 (2017).
2. Karl Pearson F.R.S.. LIII. On lines and planes of closest fit to systems of points in space. The London, Edinburgh, and Dublin Philosophical Magazine and Journal of Science, 2(11), 559–572 (1901).
3. Berman, H. M. et al. The protein data bank. Nucleic acids research, 28, 235–242 (2000).
